# Debao Wang, the founder of nucleic acid biology and industry in People’s Republic of China

**DOI:** 10.1007/s13238-020-00777-9

**Published:** 2020-08-17

**Authors:** Zhongliang Ma, Xianyi Wang, Yanli Li

**Affiliations:** Lab for Noncoding RNA & Cancer, School of Life Sciences, Shanghai University, Shanghai, 200444 China

Dr. Debao Wang (王德宝) was born on May 7th, 1918, in Taixing, Jiangsu Province, China. At the age of 18, he was admitted to the Agricultural Chemistry Department of the National Central University, and graduated in 1940. In the summer of 1943, Dr. Wang worked in the Department of Biochemistry, School of Biological Medicine, under the supervision of Prof. Libin T. Cheng (Zhang, 2020). At that time, he had a dream to contribute to the development of his motherland, which inspired him to study aboard.

**Figure 1:**
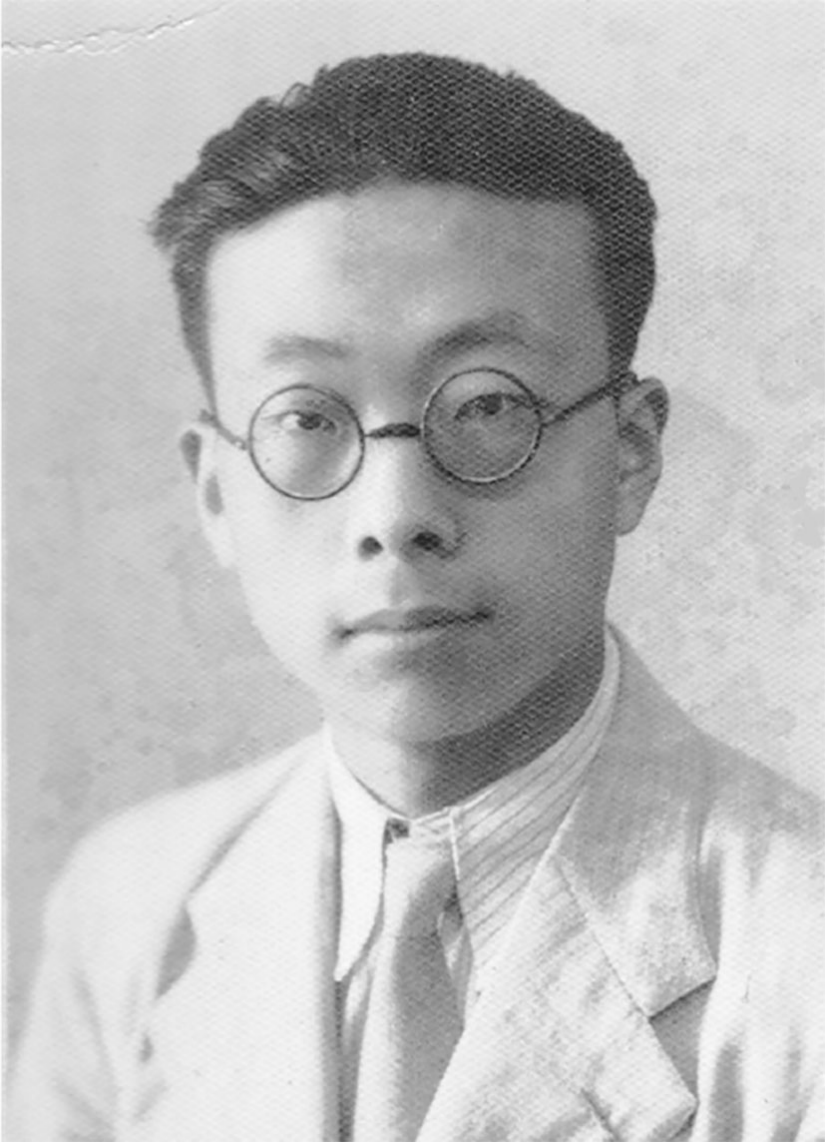
Debao Wang in the USA

In early 1947, he left to study at Louisiana State University, USA (Fig. 1). In the autumn of 1948, he studied at the Department of Biochemistry, University of Washington, under the supervision Prof. C. Cori, who won the Nobel Prize in Physiology or Medicine. In the summer of 1949, he moved to the Western Reserve University with Dr. Coci, where he received his doctorate in the fall of 1951. In the same year, Dr. Wang started his postdoctoral research at Johns Hopkins University. During his study in the United States, Dr. Wang pioneered the synthesis of nicotinamide adenine dinucleotide phosphate (NADP) from NAD, which was later used by major pharmaceutical companies in the world for 30 years. His industrial methods greatly reduced the price of NADP.

In addition, he also discovered nucleoside hydrolase, cytosine deaminase, uracil oxidase and several other new enzymes, which had a profound impact on biochemistry.

**Figure 2:**
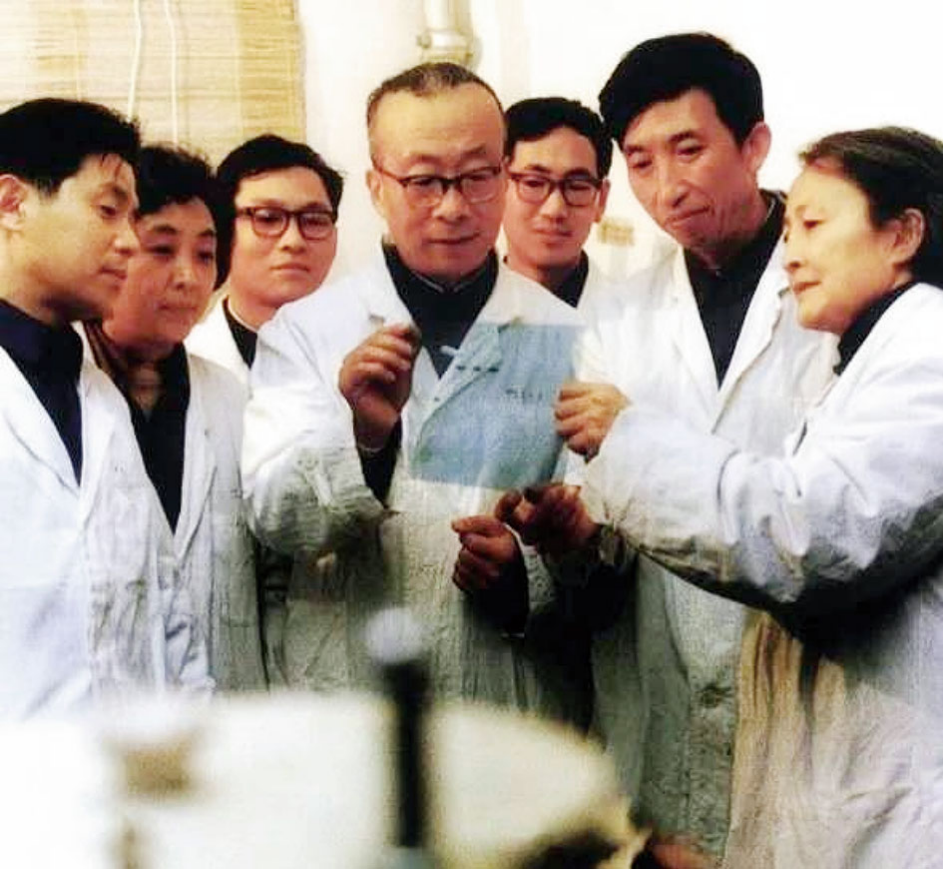
Debao Wang and his group discussing an experiment in Shanghai

In 1955, he decided to return and devote his life to scientific research in his motherland, China. He worked at the Institute of Physiology and Biochemistry of Chinese Academy of Sciences (CAS), and established the first nucleic acid research group in China. In 1961, Dr. Wang served as the director of a nucleic acid research laboratory (Fig. 2). What’s more, Dr. Wang focused on the structure and function of transfer RNA (tRNA), starting nucleic acid research in China.

Dr. Wang also used his knowledge to produce some things people needed. He and his students modified the production procedures of 4 kinds of 5′-ribonucleotides using the autolysis method, and obtained the flavor enhancer monosodium glutamate in cooperation with an MSG factory. Their work won the 1978 National Science and Technology Achievement Award and made him the founder of the production of flavor enhancers in China. Due to his great contributions to biochemistry, he was elected a member of the Chinese Academy of Sciences in 1980.

In addition, Dr. Wang investigated the synthesis of yeast tRNA^Ala^. After 13 years of unremitting efforts, his team finally completed the artificial total synthesis of yeast tRNA^Ala^ in mid-November 1981. This was the first artificial synthetic tRNA in the world, and they put a lot of efforts into it due to the complexity of RNA structures (Wang, 1983a, b; Liu, 2002). Because the second carbon of ribose in RNA contains a hydroxyl group, it is necessary to protect the group to reduce unwanted reactions. In order to solve this difficult problem, they chose to synthetize 3′-half and 5′-half tRNA, respectively, and finally connected these two halves to a complete yeast tRNA^Ala^.

They proposed that the synthetic yeast alanine tRNA has the same chemical composition (containing 9 modified nucleotides of 7 different species) and structure as the natural yeast alanine tRNA, making it biologically active (Wang, 1983a). A comparison of the charging activities of yeast alanyl tRNA and reconstituted natural yeast tRNA revealed that the anticodon loop of yeast alanyl tRNA was not involved in the interaction between alanyl-tRNA synthetase from rat liver and yeast alanyl-tRNA (Jin, 1987). This work has won the first prize of science and technology achievements of the Chinese Academy of Sciences, the first prize of the National Natural Science Foundation, and the Tan Kah Kee Award in Life Sciences. In 1983, he made a report to the 10th International tRNA Symposium on the topic of “Artificial Total Synthesis of Yeast tRNA^Ala^” which won unanimous attention and praise at home and abroad. The study of these analogues revealed the link between biological replication, translation, and metabolic pathways, providing a valuable tool for exploring the evolutionary process of life (Chen, 2000).

After the completion of yeast alanine tRNA synthesis, Dr. Wang led his team to unmask the structure and function of yeast alanine tRNA. Not only did they clarify the biological functions of yeast alanine tRNA components, but also further studied the mechanism of tRNA generation as well as its function in transporting amino acids.

In addition, Dr. Wang made great contributions to nucleic acid research and teaching talents in China. Many students, including young university undergraduates and post-graduates, have grown up quickly under his guidance and have become the backbone of nucleic acid research and teaching in China. In 1961, the Institute of Biochemistry held the first national biochemical training course. He made great efforts to compile the first nucleic acid lecture in China, “Nucleic Acids-Structure, Function, and Synthesis,” which had a profound impact on biochemistry education and research in China.

Dr. Wang passed away at the age of 84 on November 1st, 2002. He was deeply mourned by scholars at home and abroad. Professor Boliang Li, director of the Institute of Biochemistry and Cell Biology, a member of Chinese Academy of Sciences, highly praised him with the words: “Whether it is learning or being a human being, Dr. Wang is an academic model and an example for the younger generations.” Professor Youxin Jin, the director of State Key Laboratory for Biochemistry and Molecular Biology, remembered him with the words: “The kindness of teacher Wang is hard to forget, and his qualities will be inherited forever.” Our lab is studying the role of tRNA-derived fragments (tRF) in cancer, which was Professor Jin’s idea, but an update of an original tRNA research idea of Dr. Wang. In recent years, we made progress on tRF, including a patent and a number of published papers.

Dr. Wang loved his country deeply and dedicated his life to scientific research, which had a significant effect on the development of life sciences in China. Under the guidance of Dr. Wang and his team, the structure and function of tRNAs were explained and the research on noncoding RNAs was greatly promoted. He led us into a new field of RNA research. Above all, Dr. Wang left us a great deal of valuable spiritual wealth that is worthy of being learned and inherited by our younger generations forever.
